# Hydrophilic Surface Modification of Amorphous Hydrogenated Carbon Nanocomposite Films via Atmospheric Oxygen Plasma Treatment

**DOI:** 10.3390/nano13061108

**Published:** 2023-03-20

**Authors:** Algirdas Lazauskas, Mindaugas Andrulevičius, Brigita Abakevičienė, Dalius Jucius, Viktoras Grigaliūnas, Asta Guobienė, Šarūnas Meškinis

**Affiliations:** Institute of Materials Science, Kaunas University of Technology, K. Baršausko 59, LT51423 Kaunas, Lithuania

**Keywords:** oxygen plasma, hydrophilic, wetting, surface modification, DLC, DLC:SiO_x_

## Abstract

Herein we investigated hydrophilic surface modification of SiO_x_ containing amorphous hydrogenated carbon nanocomposite films (DLC:SiO_x_) via the use of atmospheric oxygen plasma treatment. The modified films exhibited effective hydrophilic properties with complete surface wetting. More detailed water droplet contact angle (CA) measurements revealed that oxygen plasma treated DLC:SiO_x_ films maintained good wetting properties with CA of up to 28 ± 1° after 20 days of aging in ambient air at room temperature. This treatment process also increased surface root mean square roughness from 0.27 nm to 1.26 nm. Analysis of the surface chemical states suggested that the hydrophilic behavior of DLC:SiO_x_ treated with oxygen plasma is attributed to surface enrichment with C–O–C, SiO_2_, and Si–Si chemical bonds as well as significant removal of hydrophobic Si–CH_x_ functional groups. The latter functional groups are prone to restoration and are mainly responsible for the increase in CA with aging. Possible applications of the modified DLC:SiO_x_ nanocomposite films could include biocompatible coatings for biomedical applications, antifogging coatings for optical components, and protective coatings to prevent against corrosion and wear.

## 1. Introduction

Diamond-like carbon (DLC) films are a class of materials that exhibit unique properties, including high hardness [1], low friction [2,3], and excellent chemical resistance [4]. These properties make DLC films attractive for a wide range of applications, such as protective coatings for medical implants [5,6,7], wear-resistant coatings for mechanical components [8,9], and anti-reflective coatings for optical devices [10,11].

The wetting properties of DLC films, or the ability of a liquid to spread over the surface and form a uniform and stable contact angle is a very important factor in determining the performance and suitability of DLC films for specific applications. Good wetting properties can enhance the functionality of DLC films in various applications, such as in medical implants, where wetting properties play a crucial role in controlling the interaction between the implant and the surrounding biological fluids [12]. DLC films with good wetting properties can exhibit improved performance in specific applications, such as in optical devices, where a uniform and stable contact angle can help reduce reflections and increase optical transmission. Furthermore, hydrophilic DLC films can be effectively utilized in environments that involve liquids or high humidity [13]. Good wetting properties can improve the adhesion between DLC films and other materials, which is important in applications such as tribological coatings and protective [14,15].

The surface energy of DLC films is an important parameter that determines their wetting and adhesion characteristics [16]. One approach to tailor the surface energy of DLC films is through the use of plasma treatment. This treatment can be easily applied and does not require hazardous chemicals or specialized equipment, making it a cost-effective and scalable method. Plasma treatment involves exposing the DLC film to gas plasma, which results in the formation/recombination of functional groups on the surface of the film. These functional groups can alter the surface energy of the DLC film and improve its wetting and adhesion properties. For example, oxygen plasma treatment has been reported to be an effective method to increase hydrophilicity as well as hemocompatibility of DLC films [17,18]. The formation of oxygen-related functional groups on the surface of the film after exposure to oxygen plasmas is mainly responsible for the increase in the hydrophilic character and surface energy of DLC [19,20].

Another strategy to improve wetting properties of DLC is through the use of hybrid films. Hybrid films are composed of DLC and other materials, such as metallic or organic compounds, which can affect the surface energy of the film. By carefully selecting the composition of the hybrid film, it is possible to achieve desired surface energy levels for specific applications, such as anti-fog coatings on automobile windshields, and biocoatings on contact lenses [21,22].

In addition to plasma treatments and hybrid films, the surface wetting properties of DLC films can be also tailored through other methods, e.g., chemical (piranha treatments [23]), photochemical (UV light and 30% hydrogen peroxide), ion implantation [24] and laser treatment [25]. These methods can also introduce functional groups onto the surface of the DLC film and modify its wetting characteristics. However, chemical treatment methods can be hazardous due to the highly reactive nature of the chemicals involved, which can pose safety concerns. Moreover, chemical modification methods may result in uncontrolled etching of the DLC film, leading to a reduced film thickness and surface roughness, which can negatively affect the DLC film properties. The photochemical method may not be effective for thicker films, and the process may require longer exposure times to achieve desired surface modification. Moreover, photochemical treatment may cause damage to the DLC film structure, leading to reduced film quality. Ion implantation and laser treatment can be expensive and require specialized equipment, which may not be easily accessible. Furthermore, these treatments may cause localized damage to the film and may not be effective for modifying the entire surface uniformly.

One specific type of DLC film—SiO_x_ containing amorphous hydrogenated carbon nanocomposite film (DLC:SiO_x_), which is widely known for its high-hardness (10–20 GPa), low wear rate (10^−5^–10^−8^ mm^3^ N^−1^ m^−1^) and friction coefficient (0.02–0.2), as well as low internal stresses (<1 GPa) and high optical transmittance (~80–85%) in the visible spectrum [11,26,27,28]. However, very little is known about the surface modification of DLC:SiO_x_ films with plasma techniques. Herein, we attempt to contribute to this topic by investigating how the wetting properties of DLC:SiO_x_ films are affected upon the atmospheric oxygen plasma treatment. We established the possible correlations between the oxygen plasma treatment and the changes it introduces to the surface of the DLC:SiO_x_ film via the use of water droplet contact angle (CA) measurements, X-ray photoelectron spectroscopy (XPS), and atomic force microscopy (AFM). It was found that atmospheric oxygen plasma treatment of the DLC:SiO_x_ film modifies its surface to be effectively hydrophilic. The wetting properties of DLC:SiO_x_ film deteriorate to some extent with aging in ambient air at room temperature.

## 2. Materials and Methods

A commercial high-grade extra clear float glass Pilkington Microwhite^TM^ (Sheet Glass Co., Tokyo, Japan) was used as a substrate material. Hexamethyldisiloxane (HMDSO) of analytical grade (≥99%, Sigma-Aldrich, Saint Louis, MO, USA) was used as a source of hydrocarbons, silicon, and oxygen for synthesis of DLC:SiO_x_ films. Deionized (DI) water with a resistivity higher than 18.2 MΩ/cm at 25 °C was used for CA measurements, and was obtained from a Direct-Q^®^ 3 UV water purification system (Merck KGaA, Darmstadt, Germany).

**Scheme 1:**
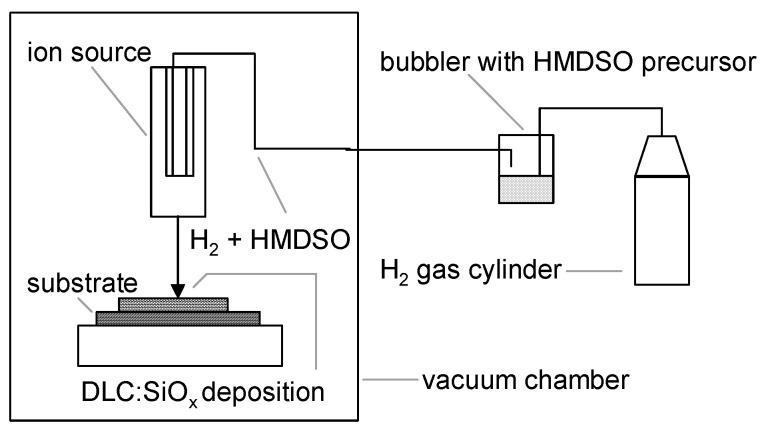
Simplified schematic illustrating experimental setup for DLC:SiO_x_ deposition.

The Hall-type closed drift ion beam source operating at a constant energy of 800 eV and a current density of 100 μA/cm^2^ was used for deposition of DLC:SiO_x_ films at room temperature. The base pressure and work pressure in the vacuum chamber were 2 × 10^−4^ Pa and 2 × 10^−2^ Pa, respectively. Hydrogen gas (H_2_) was used for transportation of HMDSO vapor into the vacuum chamber. Simplified schematic illustrating experimental setup for DLC:SiO_x_ deposition is shown in Scheme 1.

Control tests with monocrystalline Si(1 0 0) (UniversityWafer Inc., Boston, MA, USA) substrates were performed in order to determine deposition rate of DLC:SiO_x_ for H_2_ gas. The thickness of DLC:SiO_x_ on Si(1 0 0) was determined using a laser ellipsometer Gaertner L-115 (Gaertner Scientific Corporation, Skokie, IL, USA) equipped with a He–Ne laser (wavelength of 632.8 nm). Film thickness of ~100 nm was chosen for the deposition of DLC:SiO_x_ on glass substrates.

The radio frequency capacitive plasma unit Plasma-600T (JSC Kvartz, Kuvasay, Uzbekistan) operating at a frequency of 13.56 MHz and power of 0.3 W/cm^2^ was used for surface modification of as-deposited DLC:SiO_x_ films. The atmospheric oxygen plasma treatment time was varied in the range of 1–5 min.

CA measurements were performed at room temperature using the sessile drop method. The size of the DI droplets was 5 µL, the wetting angles were recorded after 10 min for all samples. CA were determined using an active contour method based on B-spline snakes (active contours) [29]. The CA is reported as an average of five measurements at different places on the surface of each sample.

AFM experiments were performed with NanoWizardIII microscope (JPK Instruments, Bruker Nano GmbH, Berlin, Germany) equipped with V-shaped silicon cantilever (spring constant of 3 N/m, tip curvature radius of 10.0 nm and the cone angle of 20°) operating in contact mode at room temperature. Data processing was carried out using a SurfaceXplorer and JPKSPM Data Processing software (Version spm-4.3.13, JPK Instruments, Bruker Nano GmbH).

The XPS measurements were performed employing XSAM800 spectrometer (Kratos Analytical Ltd., Manchester, United Kingdom). The non-monochromatized Al Kα radiation (hν = 1486.6 eV) was used for XPS spectra acquisition. The base pressure in the analytical chamber was lower than 8 × 10^−8^ Pa. The energy scale of the system was calibrated according to Au 4f7/2, Cu 2p3/2 and Ag 3d5/2 peak positions, respectively. The C 1s, O 1s, and Si 2p spectra were acquired at the 20 eV pass energy (0.1 eV energy step), and the analyzer was in the fixed analyzer transmission (FAT) mode. Spectra were fitted using a sum of Lorentzian–Gaussian (ratio of 30:70) functions and symmetrical peak shape; while for graphitic carbon asymmetrical peak shape and 70:30 ratio was used.

## 3. Results and Discussion

**Figure 1:**
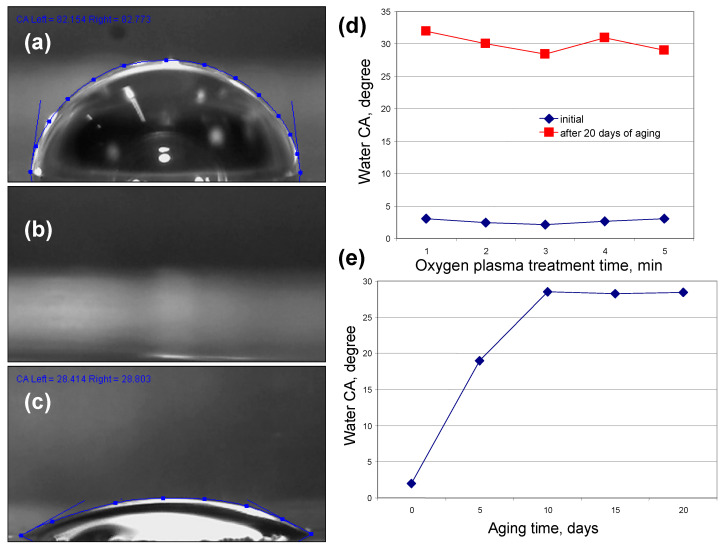
Water droplet profile images of DLC:SiO_x_ (**a**) as-deposited, (**b**) 3 min oxygen plasma treated and (**c**) oxygen plasma and aged for 20 days; (**d**) water CA as a function of oxygen plasma treatment time of as-deposited and after aging for 20 days; (**e**) water CA as a function of aging time.

Figure 1 shows typical water droplet profile images of DLC:SiO_x_ films before and after oxygen plasma treatment for 3 min, as well as the plasma treated DLC:SiO_x_ film, which was aged for 20 days in ambient air at room temperature. The as-deposited DLC:SiO_x_ film exhibited water CA of 82 ± 1°, which is very close to the hydrophobic surface. After oxygen plasma treatment for 3 min, the surface of the DLC:SiO_x_ film was modified to be effectively hydrophilic with complete spreading of water droplet (CA < 2°) on the surface. In [17], DLC films deposited using benzene and diluted silane as the precursor gases were subjected to plasma treatment using various gases such as N_2_, O_2_, H_2_, and CF_4_. They found that oxygen plasma treated films exhibited the lowest water CA of 13.4 ± 1.3° as compared to other plasma gases. In contrast, our result is significantly better. Further, we assessed the wetting stability of DLC:SiO_x_. The water CA on the surface of the DLC:SiO_x_ film, which was modified using oxygen plasma treatment increased up to 28 ± 1°, still maintaining good hydrophilic properties after 20 days of aging. It was also found that the oxygen plasma treatment time variation (i.e., 1–5 min) of as-deposited DLC:SiO_x_ films had little effect on the hydrophilic surface modification as in all cases total surface wetting was observed (Figure 1d), whereas the lowest CA was determined for the 3 min oxygen plasma treated DLC:SiO_x_ films (CA 28 ± 1°) after 20 days of aging (Figure 1d). In Figure 1e, CA measurements indicated that during the first 10 days of aging, the oxygen plasma-treated DLC:SiO_x_ film rapidly loses its hydrophilic properties to some extent, after which stabilization is reached with CA ~28 ± 1° for the remaining 10 days of aging. S. Narayan et al. investigated oxygen plasma treatment effect on the wetting properties of DLC coatings deposited using plasma enhanced chemical vapor deposition (PECVD) technique [30]. They observed that hydrophilic properties of oxygen plasma treated DLC coatings rapidly deteriorate within 8 days of aging. Afterwards, better stability of CA with aging time was observed. However, CA values of >40° were reported for oxygen plasma treated DLC coatings in all cases after 10 days of aging.

**Figure 2:**
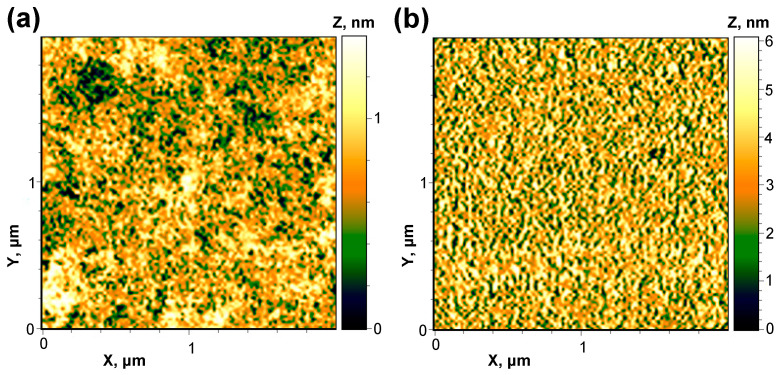
AFM surface topography of (**a**) as-deposited and (**b**) 3 min oxygen plasma treated DLC:SiO_x_ films.

Figure 2 shows characteristic AFM 2D topographical images of the as-deposited and 3 min oxygen plasma treated DLC:SiO_x_ films acquired over 2.0 × 2.0 µm^2^ area in air using contact mode. The topography of the as-deposited DLC:SiO_x_ surface exhibits a random distribution of surface mounds having different angle orientations to each other, without a preferred direction. A mean height of the surface structures (*Z*_mean_) was determined to be 0.7 nm. The root mean square roughness (*R_q_*) was found to be 0.27 nm. The as-deposited DLC:SiO_x_ film surface is dominated by the valleys with skewness (*R_sk_*) value of −0.11 and has a platykurtoic distribution (i.e., relatively few high peaks and low valleys) of surface morphological features with kurtosis (*R_ku_*) value of 2.7. In contrast, 3 min oxygen plasma treated DLC:SiO_x_ film surface exhibited higher *R_q_* value of 1.26 nm with *Z*_mean_ value of 3.23 nm, and followed similar distribution of surface morphological features with *R_sk_* and *R_ku_* values of −0.11 and 2.46, respectively. In [31,32] surface morphological analysis was performed for as-deposited and oxygen plasma treated DLC films. Their results also indicated an increase in surface roughness after surface modification with oxygen plasma. This increase in surface roughness was attributed to oxygen ion bombardment during the treatment process. In our case, oxygen plasma treated DLC:SiO_x_ film maintained very low surface roughness, and therefore it is suggested that this change had negligible effect on the wetting properties.

**Figure 3:**
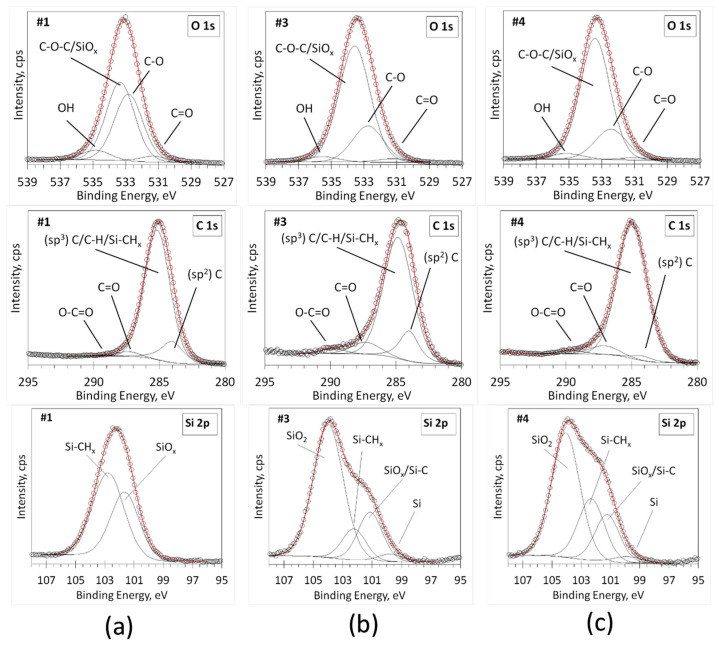
High-resolution deconvoluted XPS spectra in O 1s, C 1s and Si 2p regions of DLC:SiO_x_ (**a**) as-deposited, (**b**) 3 min oxygen plasma treated and (**c**) oxygen plasma treated and aged for 20 days.

The effect of oxygen plasma surface hydrophilic modification on the chemical states of DLC:SiO_x_ was investigated employing XPS. The deconvoluted high-resolution XPS spectra in the O 1s, C 1s and Si 2p regions of the as-deposited, oxygen plasma treated and 20 days aged DLC:SiO_x_ films are shown in Figure 3. The deconvoluted components of DLC:SiO_x_ films in the XPS O 1s spectra where assigned to C=O (531.2 eV), C–O (532.6 eV), C–O–C/SiO_x_ (533.1 eV) and O–H (534 eV) chemical bonds [33,34,35,36,37]. It can be seen that after oxygen plasma treatment the concentration of hydrophilic C–O–C functional groups on the surface of DLC:SiO_x_ increased considerably, remained stable after 20 days of aging. The deconvoluted component originating from C–O bonds decreased after oxygen plasma treatment and also remained stable after 20 days of aging. High-resolution XPS spectra in the C 1s region were deconvoluted into four components, respectively. A high intensity peak at 285 eV represents carbon in sp^3^ hybridization, and it overlaps with C–H and Si–CH_x_ chemical bonds [38]. A low intensity peak at 284.1 eV was assigned to carbon sp^2^ hybridization. The position and asymmetric shape of this component is typical for graphitic carbon [39,40]. Lower intensity peaks at higher binding energies could be assigned to O–C=O and C=O chemical bonds. No considerable changes were observed in C 1s region for oxygen plasma treated DLC:SiO_x_ films. The aging of the DLC:SiO_x_ films resulted in further oxidation in ambient air, which is indicated by slight increase of C=O component as well as recombination of sp^2^ carbon into other functional groups. Two deconvoluted components of as-deposited DLC:SiO_x_ film in the XPS Si 2p spectrum were assigned to Si–CH_x_ (102.5 eV) and SiO_x_ (101.1 eV) chemical bonds [38,41]. Two additional components appeared at 104 eV and 99.8 eV after oxygen plasma treatment of DLC:SiO_x_, assigned to the SiO_2_ and Si–Si chemical bonds, respectively [41]. After oxygen plasma treatment the concentration of hydrophobic Si–CH_x_ functional groups on the surface of DLC:SiO_x_ decreased significantly, and slightly increased after 20 days of aging, which is in good agreement with CA measurement results. The restoration of Si–CH_x_ chemical bonds is mainly responsible for CA increase with aging of DLC:SiO_x_ film. The appearance of SiO_2_ and Si–Si chemical bonds for oxygen plasma treated DLC:SiO_x_ films also significantly contributed to the effective hydrophilic properties of the surface [21]. The concentration of these functional groups on the surface of oxygen plasma treated DLC:SiO_x_ film remained relatively stable after 20 days of aging.

Based on the findings of this study, several future directions are of the main importance:

- Investigation of the long-term stability of hydrophilic properties of the oxygen plasma treated DLC:SiO_x_ films under various environmental conditions, such as humidity, temperature, and exposure to different chemicals.
- Study of the underlying mechanisms of the restoration of hydrophobic Si-CH_x_ functional groups and the ways to prevent or delay this process, enhancing the long-term stability of the hydrophilic properties of the DLC:SiO_x_ films.
- Investigation of the effect of the hydrophilic DLC:SiO_x_ films on the adhesion, proliferation, and differentiation of various cell types to explore their potential applications in tissue engineering and regenerative medicine.

## 4. Conclusions

Atmospheric oxygen plasma treatment was used to modify DLC:SiO_x_ film wetting properties. The surface of the DLC:SiO_x_ film was modified to be effectively hydrophilic with complete spreading of water droplet (CA < 2°) on the surface. The CA increased up to 28 ± 1° after 20 days of aging in ambient air at room temperature, still maintaining good hydrophilic properties. AFM analysis indicated that the root mean square roughness of the film increased from 0.27 nm to 1.26 nm after oxygen plasma treatment. XPS investigation revealed that the highly hydrophilic characteristics of the oxygen plasma treated DLC:SiO_x_ is attributed to surface enrichment with C–O–C, SiO_2_ and Si–Si chemical bonds as well as significant removal of hydrophobic Si–CH_x_ functional groups. During aging process of DLC:SiO_x_ film the Si–CH_x_ functional groups tend to restore to some extent negatively affecting the wetting properties. The modified DLC:SiO_x_ nanocomposite films should be tested in various applications, such as biocompatible coatings for medical purposes, anti-fog coatings for optical components, and protective coatings to prevent corrosion and wear.

## Data Availability

Not applicable.
